# On the Futility of Screening for Genes That Make You Fat

**DOI:** 10.1371/journal.pmed.1001114

**Published:** 2011-11-01

**Authors:** J. Lennert Veerman

**Affiliations:** School of Population Health, The University of Queensland, Brisbane, Queensland, Australia

## Abstract

J. Lennert Veerman discusses the implications for genetic screening of findings showing that physical activity substantially attenuates the effects of genetic variants which predispose towards obesity.

**Linked Research Article**

This Perspective discusses the following new study published in *PLoS Medicine*:

Kilpeläinen TO, Qi L, Brage S, Sharp SJ, Sonestedt E, et al. (2011) Physical Activity Attenuates the Influence of *FTO* Variants on Obesity Risk: A Meta-Analysis of 218,166 Adults and 19,268 Children. PLoS Med 8(11): e1001116. doi:10.1371/journal.pmed.1001116

Ruth Loos and colleagues report findings from a meta-analysis of multiple studies examining the extent to which physical activity attenuates effects of a specific gene variant, *FTO*, on obesity in adults and children. They report a fairly substantial attenuation by physical activity on the effects of this genetic variant on the risk of obesity in adults.

An impressively large meta-analysis in this issue of *PLoS Medicine* shows that physical activity modifies the effect of a common genetic trait on body mass. It was already known that certain variants of the “fat mass and obesity associated” (*FTO*) gene predispose to weight gain, but this article shows that this effect is weaker among physically active persons [1].

The authors argue that this is important because many people have a determinist view of genes and may think that when something is written in your genetic code, nothing can be done to alter the course of fate. The study shows that view to be overly simplistic: genes may predispose to weight gain, but this weight can be lost by extra physical activity. And in time, studies into the causes of this gene–behaviour interaction may lead to new treatments for obesity.

## Limited Public Health Relevance

Encouraging as that news is, the immediate relevance of this study for public health is limited. The logical consequence of genomic research is screening. Genetic screening for obesity is already commercially available. The results tell the customer their lifetime risk of obesity and how much that differs from the population average. The validity of such direct-to-consumer genomic tests is largely unknown [2], and before rushing to screening programmes, some critical reflection on the role and risks of genetic screening for susceptibility to behavioural risk factors is warranted. There are at least four reasons why screening individuals for genes that predispose to obesity makes little clinical sense and may even do harm. Genetic screening for obesity has limited predictive power, is unlikely to inform therapeutic decisions, does not add to body mass index (BMI) as predictor of disease, and may distract from the societal changes that most experts think are needed to reduce the prevalence of obesity.

## Weak Predictive Power

First, individual genetic traits do not seem to have all that much influence on body mass. The impact of genetic traits on population health is a product of the size of the health effect for the affected individual (penetrance) and the frequency of the trait in a population. The rs9939609-variant of the *FTO* gene studied by Kilpeläinen et al. is common, but although it is the strongest known susceptibility locus for common obesity, its penetrance is low. A single copy is associated with a 23% increased risk of obesity and a correspondingly modest effect on body mass of 0.36 kg/m^2^ (about 1 kg) on average. Modelling studies have shown that even when testing for multiple genetic traits with such low predictive power, screening is unlikely to be worthwhile [3],[4]. And since genetic traits with the highest predictive power are most likely to be the first ones found, it is unlikely that genetic traits with larger impact on body mass will ever be found.

## No Change in Therapeutic Options

Second, testing for genetic traits that are associated with obesity makes no difference in the advice to overweight persons: increased physical activity and a healthy diet are indicated regardless of the genes. If the results of genetic testing would motivate and empower persons to do better than average [5], such testing might add value, but with equal right one may speculate that others could feel discouraged to improve health behaviours. Either way, beyond the suggestion that genomic screening may spur further screening for early stages of disease, the evidence on how genetic testing influences health behaviours remains largely anecdotal and speculative [2],[6]. Theoretically, genomic profiling might indicate the most efficient way to lose weight and reduce the risk of disease, but given the limited accuracy with which we can measure diet and (until recently) physical activity levels, it may take a while to develop clinically relevant prediction models.

## No Better Prediction of Disease

The third and most fundamental reason for the futility of screening for obesity-enhancing genetic traits is that it adds no predictive value to existing disease prediction tools. This is perhaps best illustrated in the original study that reported on the significance of *FTO* for obesity. Frayling et al. wrote that “the association between *FTO* SNPs and type 2 diabetes was abolished by adjustment for BMI, which suggests that the association of these SNPs with T2D risk is mediated through BMI” [7]. Of course, a test that adds no predictive power to a simple measurement of BMI is not worth doing.

## Wrong Focus

Fourth, screening raises some further critical issues at the societal level, notably that “a focus on genetic susceptibilities may be used to shift the focus of public health intervention and policy to the individual level and away from larger social, economic, and political factors that are fundamental to the production of human health and illness” [8]. This criticism is very relevant in the case of obesity, as many obesity experts argue that the obesity epidemic is related exactly to those larger social, economic, and political factors (the “obesogenic” environment) [9],[10]. Genes may co-determine who becomes obese, but our environment determines how many become obese. With the current state of technology, apart from bariatric surgery the only solution for the obesity epidemic lies in changes to the environment so that it promotes physical activity and a healthy diet. A focus on individual genetic traits is a mere distraction and reinforces the popular view of obesity as a problem that individuals have to deal with, rather than one that requires societal action.

## Abbreviations

BMI: body mass index
